# De novo production of resveratrol from glycerol by engineering different metabolic pathways in *Yarrowia lipolytica*

**DOI:** 10.1016/j.mec.2020.e00146

**Published:** 2020-09-19

**Authors:** Qin He, Patrycja Szczepańska, Tigran Yuzbashev, Zbigniew Lazar, Rodrigo Ledesma-Amaro

**Affiliations:** a Department of Bioengineering and Imperial College Centre for Synthetic Biology, Imperial College London, London, SW7 2AZ, UK; b Department of Microbiology, Key Lab of Microbiological Engineering of Agricultural Environment, Ministry of Agriculture, College of Life Sciences, Nanjing Agricultural University, Nanjing, 210095, PR China; c Department of Biotechnology and Food Microbiology, Wroclaw University of Environmental and Life Sciences, Chelmonskiego 37, 51-630, Wroclaw, Poland

**Keywords:** Resveratrol, *Yarrowia lipolytica*, Metabolic engineering, Golden gate, Tyrosine pathway, Phenylalanine pathway

## Abstract

Resveratrol is a polyphenol with multiple applications in pharma, cosmetics and food. The aim of this study was to construct *Yarrowia lipolytica* strains able to produce resveratrol. For this purpose, resveratrol-biosynthesis genes from bacteria and plants were expressed in this host. Since resveratrol can be produced either via tyrosine or phenylaniline, both pathways were tested, first with a single copy and then with two copies. The phenylalanine pathway resulted in slightly higher production in glucose media, although when the media was supplemented with amino acids, the best production was found in the strain with two copies of the tyrosine pathway, which reached 0.085 ​g/L. When glucose was replaced by glycerol, a preferred substrate for bioproduction, the best results, 0.104 ​g/L, were obtained in a strain combining the expression of the two synthesis pathways. Finally, the best producer strain was tested in bioreactor conditions where a production of 0.43 ​g/L was reached. This study suggests that *Y. lipolytica* is a promising host for resveratrol production from glycerol.

## Highlights

• Resveratrol can be produced in engineered Y. lipolytica via Tyrosine or Phenylalanine.
• The integration of multiple copies of the pathway genes further increased production.
• Engineered Y. lipolytica can produce 430 ​mg/L of resveratrol from glycerol.

## 1 Introduction

Resveratrol (trans-3,5,4′-trihydroxystilbene), a natural polyphenolic compound of the stilbene class, is naturally found in grapes, blueberries, peanuts, etc., and their processed products (Li et al., 2015). Resveratrol showed beneficial effects in various preclinical test (Jeandet et al., 2012; Mei et al., 2015), aimed to provide benefits against tumor, inflammation, diabetes, thrombosis and aging. Therefore, resveratrol has gained an increased interest by pharmaceutical, food and cosmetic industries. At present, the major source of commercial resveratrol is plant extracts such as Japanese knotweed *Polygonum cuspidatum* (Mei et al., 2015) and grapes.

**Fig. 1:**
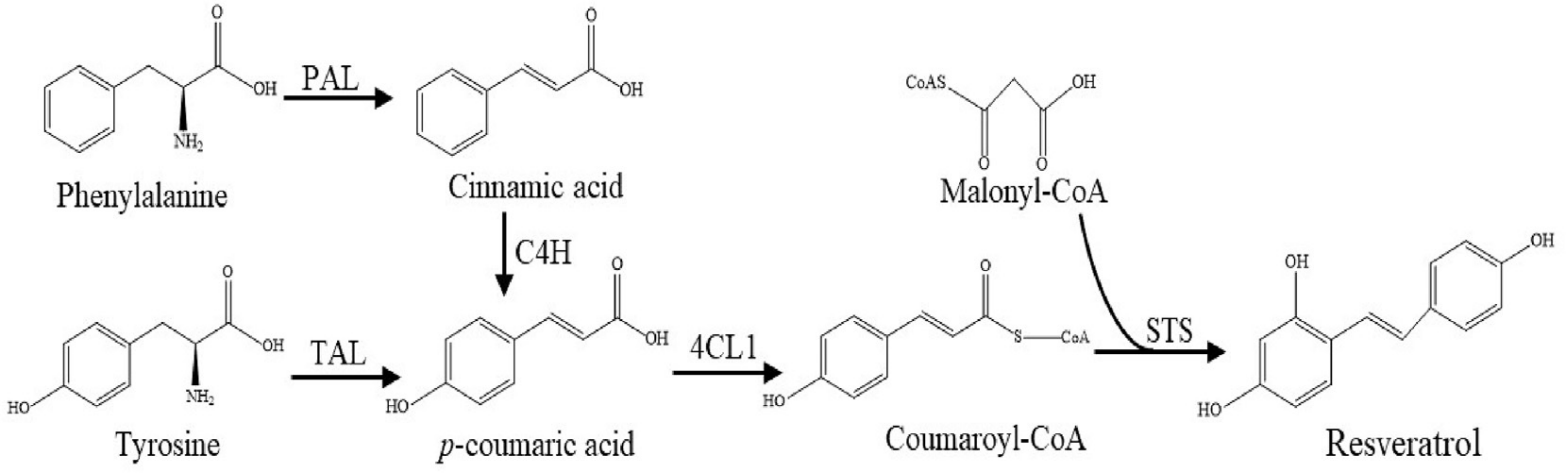
Biosynthesis paths for resveratrol. PAL: Phenylalanine ammonia lyase; C4H: Cinnamic acid 4-hydroxylase; TAL: Tyrosine ammonia lyase; 4CL1: 4-coumaroyl-CoA ligase; STS: Stilbene synthase.

Biotechnological production by recombinant strains provides a potential approach for resveratrol biosynthesis (Borodina and Nielsen, 2014). As shown in Fig. 1, there are two main pathways for resveratrol production, one using tyrosine and the other phenylalanine as intermediates. For the tyrosine pathway, first, a tyrosine ammonia lyase (TAL) deaminates L-tyrosine to *p*-coumaric acid. Then a 4-Coumarate: CoA ligase (4CL) forms coumaroyl-CoA from coumaric acid. Finally, coumaroyl-CoA is condensed by three malonyl-CoA units to form resveratrol by a stilbene synthase (STS) (Wu et al., 2017). On the other hand, through the phenylalanine pathway, first, cinnamic acid is generated by deamination of phenylalanine via a phenylalanine ammonia lyase (PAL). Next, cinnamic acid is hydroxylated to *p*-coumaric acid by a cinnamic acid 4-hydroxylase (C4H). Finally, similarly as in the tyrosine pathway, cinnamic acid can be converted in resveratrol via 4CL and STS.

Presently, most research aimed to produce resveratrol in heterologous host has focused on synthesis via tyrosine (Shin et al., 2012) and mainly in recombinant *E. coli* and *Saccharomyces cerevisiae* (Li et al., 2015). Resveratrol produced through tyrosine by recombinant *S. cerevisiae* reached 531 ​mg/L resveratrol (Li et al., 2015) while via phenylalanine reached 812 ​mg/L resveratrol in fed-batch fermentation from glucose (Li et al., 2016).

*Yarrowia lipolytica* is an oleaginous yeast with high industrial potential (Larroude et al., 2018; Ledesma-Amaro and Nicaud, 2016a; Wang et al., 2020; Xu et al., 2020). It provides several advantages as a eukaryotic host, including its ability to utilize vast raw substrates like glycerol (Ledesma-Amaro and Nicaud, 2016b). *Y. lipolytica* is well known for its capacity to overproduce lipids which are produced from malonyl-CoA (Wang et al., 2020). Since resveratrol production requires the condensation of 3 malonyl-CoA molecules, the high availability of this molecule in *Y. lipolytica* could be advantageous to produce high titers of resveratrol. This organism has been recently engineered to produce different molecules from the shikimate pathway such as 2-phenylethanol, *p*-coumaric acid, violacein, and deoxyviolacein (Gu et al., 2020), naringenin (Lv et al., 2020; Palmer et al., 2020; Wei et al., 2020) or arbutin (Shang et al., 2020). *Y. lipolytica* has also been reported to produce small amount of resveratrol when engineered with such purpose. To our knowledge, there are four published reports on the production of resveratrol by *Y. lipolytica.* By introducing the genes downstream of L-phenylalanine, 1.46 ​mg/L of resveratrol was obtained in minimal medium supplemented with 2 ​mM tyrosine (Huang et al., 2010). In another report, 1) after relieving the feedback regulation of the shikimate pathway by eliminating amino acids formation and engineering feedback-insensitive DAHP synthases, 2) overexpressing of heterologous phosphoketolase and 3) deleting of pyruvate kinase, 12.67 ​mg/L of resveratrol was produced from tyrosine (Gu et al., 2020). Also from tyrosine, Palmer et al. (2020) produced 8.8 ​mg/L resveratrol de novo, exploiting beta-oxidation as a source of malonyl CoA. The titer increased to 48.7 ​mg/L when 2 ​mM-coumaric acid was supplemented. During the review process of this article, Sáez-Sáez et al. (2020) reported a resveratrol titer of 52.1 ​mg/L by a strain harboring FjTAL. After engineering with feedback-insensitive alleles of *ARO4* and *ARO7*, and with five additional copies of the heterologous biosynthetic genes*,* resveratrol production reached 409.0 ​mg/L in batch and 12.4 ​g/L in fed-batch.

In this work, we have created resveratrol-producing strains of *Y. lipolytica* exploiting both, the tyrosine and the phenylalanine branches of the pathway. Further optimization was achieved via the expression of multiple copies of the relevant genes as well as with combinations of the two biosynthesis pathways. Finally, the production from the preferred industrial substrate, glycerol was evaluated in bioreactor.

## 2 Materials and methods

### 2.1 Strains and culture conditions

The NEB® Turbo Competent *Escherichia coli* cells were used for all the cloning and plasmid propagation work. The *E. coli* transformants were selected and maintained on plates, and grown on broth of LB medium containing ampicillin (100 ​μg/ml) or spectinomycin (100 ​μg/ml) at 37 ​°C. *Y. lipolytica* Pold (wt), derived from W29 (ATCC20460), was used as parental strain to construct resveratrol-producing strains. were selected on hygromycin (0.2 ​g/L) when necessary (Larroude et al., 2018). Propagation of *Y. lipolytica* strains were performed using YPD medium (see composition below). Chemical reagents were purchased from Sigma-Aldrich.

### 2.2 Genes and plasmids

*Phenylalanine ammonia lyase (PAL)* from Vitis vinifera *(GeneBank accession number*: X75967) (Dubrovina et al., 2010), cinnamic acid 4-hydroxylase (C4H) from Arabidopsis thaliana *(No. NM128601)* (Shin et al., 2012), tyrosine ammonia-lyase (FjTAL) from *Flavobacterium johnsoniae (No. KR095306.1)* (Li et al., 2015)*,* 4-coumaroyl-CoA ligase (4CL1) from *Arabidopsis thaliana (No. NM104046)* (Shin et al., 2012; Li et al., 2015), resveratrol synthase (VvVST) from *Vitis vinifera (No. NM_001281010)* (Li et al., 2015). The genes were codon-optimized for *Y. lipolytica* as in GeneArt. Constructs were complemented by pre-designed 4-nt overhangs and BsaI restriction sites based on requirements of golden-gate method (Celinska et al., 2017) and synthesized by GeneArt (Life Technologies).

All restriction enzymes were purchased from New England Biolabs (NEB). Plasmids from *E. coli* were extracted using the QIAprep Spin Miniprep Kit (Qiagen). Competent *E. coli* cells were transformed by thermal shock protocol. The correct genomic insertions of the expression cassettes in *Y. lipolytica* were verified from genomic DNA and PCR using primers listed in Table S1. Yeast transformation was carried out following lithium-acetate method (Barth and Gaillardin, 1996). Transformants were selected on YNB-Leu, YNB-Ura, or YNB-Hygro media, depending on their genotype. Plasmids used in this study are listed in Table S2.

### 2.3 Expression cassettes construction

DNA constructions

DNA cassette was constructed using the recently developed Golden Gate Assembly (GGA) method for *Y. lipolytica* (Celinska et al., 2017; Larroude et al., 2018). The synthesized genes were introduced into the donor vector pYTK001. Promoter *TEF*, terminator *Lip2*, and the pYTK001/genes mixed equimolarly were then introduced into vectors pB(Z)US1.1 (2/3) in one-pot reaction together with BsaI (0.5 ​μl), T4 DNA ligase (0.5 ​μl), T4 DNA ligase buffer (1 ​μl) and ddH_2_O up to 10 ​μl. The enzymatic reaction conditions were as follows: [42 ​°C for 2 ​min, 16 ​°C for 5 ​min] ​× ​25 cycles, 60 ​°C for 10 ​min, 80 ​°C for 10 ​min, and stored at 4 ​°C. After reaction, the mixture was used for *E. coli* transformation. White colonies were selected as the complete GGA candidates and identified through plasmid isolation, restriction digestion and colony PCR.

The successfully constructed plasmids were combined together in appropriate order to build DNA cassettes using similar enzymatic reaction system and condition except that BsmBI (0.5 ​μl) was used instead of BsaI. The ultimate GGA was subsequently linearised using NotI and transformed into *Y. lipolytica* after confirmation*.* The transformants were screened by nutrient deficient medium and verified PCR using genomic DNA.

### 2.4 Strains construction

4CL1 and STS are the common enzymes to synthesize resveratrol. In this experiment, the two pathways were constructed independently. Strain T contained *FjTAL*, *4CL1*, and *VvVST* genes to produce resveratrol from tyrosine. Strain P contained phenylalanine path coding genes *PAL*, *C4H*, *4CL1*, and *VvVST*. Intermediate strains were obtained by removing the marker from strains T and P respectively, using Cre/lox method (Fickers et al., 2003). The second copy of the expression DNA cassettes responsible for the specific path were introduced into these strains, using lithium-acetate method to study the high-expression of resveratrol. All the constructed yeast strains used in this study were listed in Table S3.

### 2.5 Resveratrol production in flasks

Two transformants of each strain were tested. The preculture was conducted in 50 ​mL of rich YPD medium at 28 ​°C with 250 ​rpm agitation for 48 ​h. After that time, cells were washed twice with sterile distilled water and used for inoculation of the production medium. An initial OD_600_ was set at 0.5 for each strain. The main culture was conducted in 50 ​mL YNB minimal medium (6.8 ​g/L yeast nitrogen base without amino acids containing 5 ​g/L of ammonium sulfate (YNBww); 20 ​g/L glucose/glycerol; and 50 ​mM phosphate buffer pH 6.8), containing 0 or 2 ​mM tyrosine and/or phenylalanine in 300 ​mL flasks. After 72 ​h cultivation at 28 ​°C with 250 ​rpm agitation, the OD_600_, dry biomass as well as concentration of the substrate and resveratrol and coumaric acid were measured. Coumaric acid and resveratrol were extracted by mixing equal volume of absolute ethanol with the culture and centrifuged at 2272 ​*g* for 30 ​min. The supernatants were used to analyze *p*-coumaric acid and resveratrol by HPLC. For glycerol as carbon source, 20 ​g/L of this compound was used instead of glucose.

### 2.6 Resveratrol production in bioreactor

The inoculum for selected strain (T2P2) was grown 48h in YPD medium at 28 ​°C, 250 ​rpm. After that time, cells were washed twice with sterile distilled water. The initial OD600 in bioreactor was set to 0.5. The cultures for T2P2 were performed in a 5-L stirred-tank bioreactor (BIOSTAT B-PLUS, Sartorius, Germany) with a working volume of 2 ​L ​at 28 ​°C. Aeration and stirring rate were set for 0.8 vvm and 800 ​rpm, respectively. The pH was maintained automatically at 6.8 by the addition of 30% NaOH solution. Five different conditions were tested using YNB medium with 100 ​g/L of glycerol as a substrate, 1.7 ​g/L of YNB and 5 ​g/L of ammonium sulfate; 1) addition of 5 ​mM of both Phe and Tyr at the beginning of the culture, 2) addition of 2 ​mM of both Phe and Tyr at the beginning of the culture, 3) without addition of amino acids, 4) lower aeration and stirring rate (0.6 vvm/500 ​rpm) with addition of 2 ​mM of both Phe and Tyr 5) limited aeration and stirring rate (0.6 vvm/500 ​rpm) without addition of amino acids. All the cultures were conducted until complete exhaustion of the carbon source. The bioreactor with appropriate medium was autoclaved at 121 ​°C, 30 ​min, 1 ​atm.

### 2.7 Analytical methods

Resveratrol and *p*-coumaric acid were quantified on HPLC (UltiMate3000, ThermoScientific) equipped with a Hypersile GOLD TM 150 ​× ​4.6 (particle size 5 ​μm). The eluent (70% acetonitrile, 0.1% formic acid) flow rate was set to 1.0 ​mL ​min^−1^. Resveratrol was detected by absorbance at 304 ​nm with a retention time of 6.4 ​min and *p*-coumaric acid at 280 ​nm of 4.7 ​min. Resveratrol and *p*-coumaric acid concentrations were calculated from the standard curves, and both resveratrol and *p*-coumaric acid standards were purchased from Sigma-Aldrich.

Dry biomass was analyzed using 10 ​mL of the culture, washed twice with sterile distilled water and filtered through 0.22 ​μm membrane. Dry biomass was analyzed gravimetrically by drying it at 105 ​°C.

## 3 Results and discussion

### 3.1 Resveratrol biosynthesis via tyrosine

**Fig. 2:**
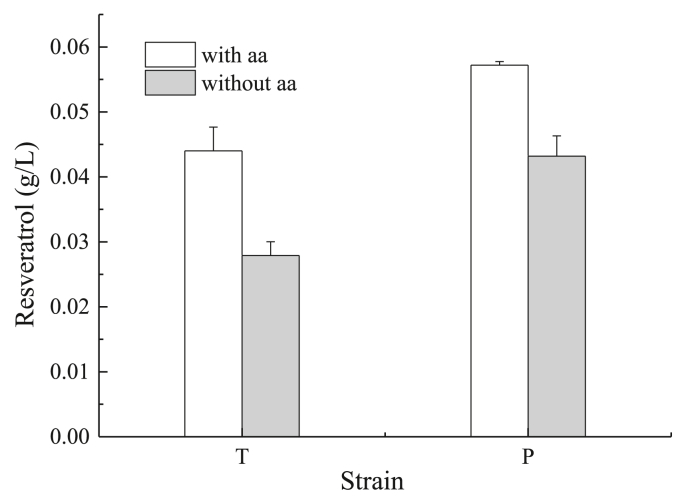
Resveratrol production by strain T and P using minimal glucose media (YNB) with and without addition of amino acids 2 ​mM of Phe and 2 ​mM of Tyr.

In order to enable the synthesis of resveratrol in *Yarrowia lipolytica*, we first overexpressed the genes involved in the conversion of tyrosine into the product of interest. The tyrosine ammonia-lyase gene from *F. johnsoniae* (*FjTAL*), the 4-coumarate:CoA ligase gene from *A. thaliana* (*4CL1*) and the stilbene synthase gene from *V. vinifera* (*VvVST*) were introduced under the control of strong and constitutive *TEF* promoter. The resulting strain (strain T) was grown in YNB glucose minimal media with or without the addition of 2 ​mM of tyrosine and phenylalanine (362 ​mg/L Tyr; 330 ​mg/L Phe). Resveratrol concentration reached 0.044 ​g/L when phenylalanine and tyrosine were added, and 0.028 ​g/L when no amino acid were added to the medium (Fig. 2). It was also noticed that in the presence of external amino acids the strain accumulated small amounts of coumaric acid (2.7 ​mg/L), which was not found in the non-supplemented media. Interestingly, we also noticed that biomass was reduced by 60% when amino acids were added to the media coinciding with a higher production of resveratrol (Supplementary Fig. S1). This could indicate that either resveratrol or other intermediates in the pathway may be toxic to the cells. Interestingly we only found weaker growth when resveratrol was added to the media at higher concentrations than 0.1 ​g/L (data not shown). Further experiments would be necessary to elucidate what is causing the reduced growth in this strain.

### 3.2 Resveratrol biosynthesis via phenylalanine

In order to create a strain able to produce resveratrol using phenylalanine as intermediate we overexpressed a phenylalanine ammonia lyase (PAL) gene from Vitis vinifera, a Cinnamic acid 4-hydroxylase (C4H) from Arabidopsis thaliana as well as the genes *4CL1* and *VvVST*. The resulted strain (strain P) was able to produce 0.043 ​g/L of resveratrol from glucose and 0.057 ​g/L when the media was supplemented with amino acids. The obtained results clearly indicate that the phenylalanine pathway for resveratrol production performs better on glucose compared to the tyrosine pathway, in both conditions (with and without amino acids supplementation; Fig. 2). According to the previous observations, higher resveratrol secretion in supplemented medium reduced the produced biomass (12% that of the P strain without supplements) (Supplementary Fig. S1). In this case, the accumulation of coumaric acid was not observed in any of the conditions, also suggesting a higher flux via phenylalanine.

To our knowledge, there is only one published report on the production of resveratrol by *Y. lipolytica* via phenylalanine (Huang et al., 2010) and three via tyrosine (Gu et al., 2020; Palmer et al., 2020; Sáez-Sáez et al., 2020). They obtained 1.46 ​mg/L from phenylalanine, and 12.67 ​mg/L, 8.8 ​mg/L and 52.1 ​mg/L from tyrosine, respectively, which is 39-fold lower than the strain P and similar levels to the strain T generated in this work. Such differences can be explained by the use of different parental strain, different genes, promoters and expression cassettes.

### 3.3 Enhanced resveratrol biosynthesis using multi-copy integration

Multi-copy integration is a strategy that has proven useful for enhancing metabolic fluxes for bioproduction in *Y. lipolytica* (Larroude et al., 2018). Therefore, here we decided to test whether further copies of the genes used to produce resveratrol either from tyrosine or phenylalanine could help to increase the metabolic flux through the pathway. Accordingly, we generated 3 new strains, T2 (with 2 copies of TAL, 4CL1 and STS), P2 (with 2 copies of PAL, C4H, 4CL1 and STS) and T2P2 (with 2 copies of PAL, C4H, TAL, 4CL1 and STS).

**Fig. 3:**
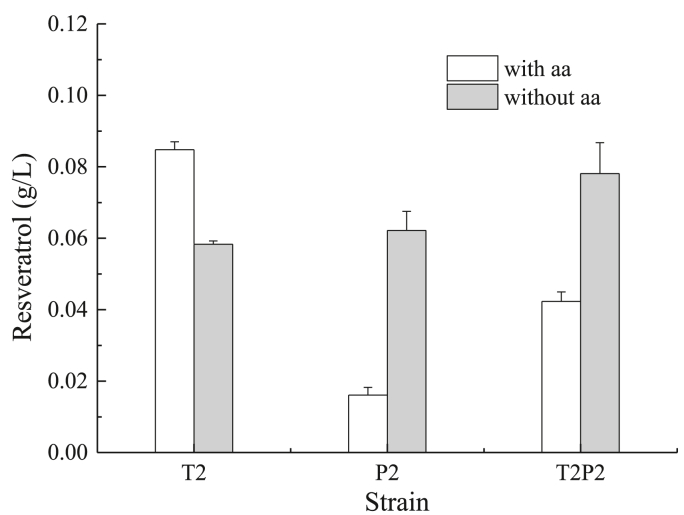
Resveratrol production by strain T2, P2 and T2P2 using glucose media with and without addition of amino acids 2 ​mM of both Tyr/Phe.

After growing the strain in the same two media as T and P (with and without amino acids supplementation), we observed that the best production in the media without amino acids was 0.078 ​g/L by the strain T2P2 while in the media with amino acids was T2 with 0.085 ​g/L (twice more than the T strain) (Fig. 3). Surprisingly, the addition of 2 copies of the genes from the phenylalanine pathway, in both P2 and T2P2, reduced significantly the production of resveratrol in media supplemented with amino acids, which was accompanied by a slight accumulation of coumaric acid, 17.9 and 21.7 ​mg/L respectively, which is an order of magnitude higher than in the T and P strains. These two strains also showed a very reduced biomass production in both media (Supplementary Fig. S2).

### 3.4 Resveratrol production from glycerol in *Y. lipolytica*

**Fig. 4:**
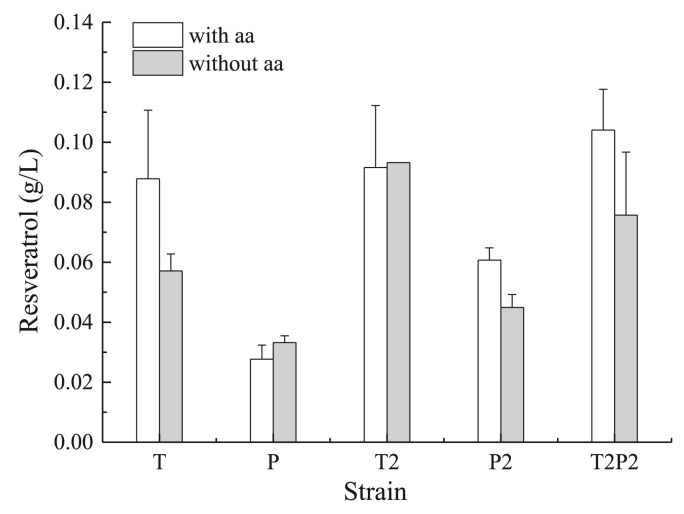
Resveratrol production by recombinant strains using glycerol as carbon source with and without addition of amino acids 2 ​mM of Phe and 2 ​mM of Tyr.

Since glycerol is a preferred substrate for biotechnology (Da Silva et al., 2009) we decided to test the capacity of the generated strains to produce resveratrol from glycerol as a sole carbon source. Wild strains of *Y. lipolytica* use glycerol very efficiently (Ledesma-Amaro and Nicaud, 2016b). Due to that, resveratrol biosynthesis from glycerol was first investigated in flask (Fig. 4).

Unlike the glucose medium, in the glycerol based medium without amino acid supplementation the strain T (0.057 ​g/L) produced higher amount of resveratrol compared to the P strain (0.033 ​g/L). The P strain also showed reduced growth using glycerol as carbon source, and, similarly to glucose, the addition of amino acids to the media further reduced biomass formation (Supplementary Fig. S3). Multi-copy integration showed better performance than single copy and T2 strain produced more than T, same as P2 produced more than P. Furthermore, T2P2 strain turned out to perform even better than T2 and P2 strains. In accordance to the results in glycerol the best performing strains were T2 and T2P2 with a production of 0.093 and 0.076 ​g/L without amino acids supplementation, 0.092 and 0.104 ​g/L with amino acids, respectively.

Summarizing this part of the study, strains T2 and T2P2 were the best strains regardless whether amino acids were added or not. We therefore selected the strain T2P2 to carry out experiments in bioreactor.

### 3.5 Resveratrol production in bioreactor

**Fig. 5:**
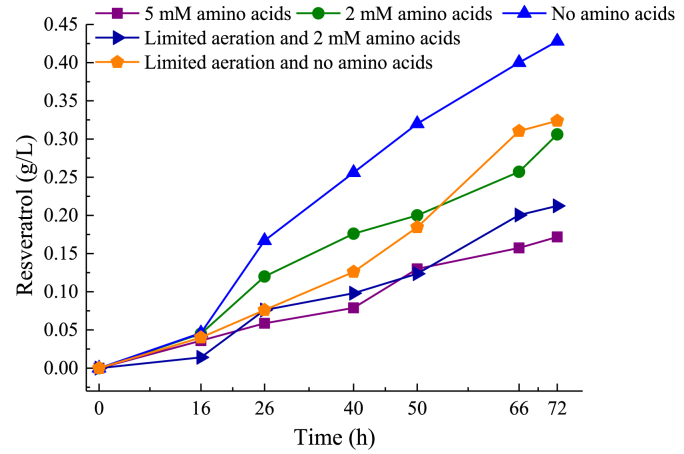
Resveratrol production by strain T2P2 using glycerol as a substrate in bioreactor.

First, we tested five conditions using 100 ​g/L of glycerol on the strain T2P2 in bioreactor; 1) addition of 5 ​mM of both Phe and Tyr at the beginning of breeding, 2) addition of 2 ​mM of both Phe and Tyr at the beginning of breeding, 3) without addition of amino acids, 4) limited aeration and stirring (0.6 vvm/500 ​rpm) with addition of 2 ​mM of both Phe and Tyr 5) limited aeration and stirring (0.6 vvm/500 ​rpm) without addition of amino acids. As shown in Fig. 5 (Supplementary Fig. S4, S5 and S6), the maximum resveratrol was produced in condition 3, where no amino acid was added, the production reached 0.43 ​g/L. In comparison, with the addition of 5 ​mM and 2 ​mM amino acids, 0.17 and 0.31 ​g/L of resveratrol were secreted, respectively (Fig. 5). The oxygen limited conditions also reduced the production titers to 0.32 ​g/L with no addition of amino acids and 0.21 ​g/L when the amino acids were added (Fig. 5).

After 72 ​h, all the glycerol was consumed for all the conditions with the exception of condition 4 (Supplementary Fig. S4). The best condition among the analyzed turned out to be condition 3, where all the substrate was consumed within 66 ​h and the produced biomass reached 18.4 ​g/L (Supplementary Fig. S6). In the worst performing condition, where only 0.17 ​g/L of resveratrol was secreted, condition 1, glycerol was consumed the fastest (within 40 ​h), however, the highest amount of biomass 40 ​g/L was also reached (Supplementary Fig. S4 and Fig. S5).

These results indicate that *Y. lipolytica* could be a good host for producing shikimate derived molecules, as it has been previously proposed for other compounds derived from aromatic amino acids (Gu et al., 2020), arbutin (Shang et al., 2020), bisdemethoxycurcumin (Palmer et al., 2020), etc. *Y. lipolytica*, with just the overexpression of the minimal set of enzymes was already able to produce de novo 430 ​mg/L of resveratrol, while similar modifications in other hosts such as *E. coli* and *S. cerevisiae* produced, for example, about 57.77 ​mg/L (Zhao et al., 2018) and 3.3 ​mg/L (Shin et al., 2012), respectively. Further production of 200 ​mg/L resveratrol was achieved by *S. cerevisiae* in batch and 415.65 and 531.41 ​mg/L in fed-batch bioreactor cultures using glucose or ethanol as substrate, respectively (Li et al., 2015). In order to achieve that they used a strain engineered to over-express feedback-insensitive alleles of *ARO4* and *ARO7*, post-translationally de-regulated acetyl-CoA carboxylase (*ACC1* gene*)*, and the integration of multiple copies of the genes of these pathways. In another report, Li et al. (2016) did further improvement in resveratrol biosynthesis by optimizing the electron transfer to the cytochrome P450 monooxygenase, increasing precursors supply, and decreasing pathway intermediates degradation, which resulted in a production of 812 and 755 ​mg/L of resveratrol in fed-batch from glucose and ethanol feed, respectively. Similar modification would likely further improve the titers that can be achieved in *Y. lipolytica* and they should be tested in the future.

## 4 Conclusions

In this work, we explored the potential of *Yarrowia lipolytica* to produce resveratrol. We found that significant production was achieved either from tyrosine or phenylalanine as precursors and we observed that the increase in the copy numbers of the genes of the pathway enhanced the synthesis of resveratrol. Interestingly, we found that the addition of the precursors tyrosine and phenylalanine to the culture broth have a negative effect in production in bioreactor. Finally, the best strain (T2P2) combining the two pathways and multiple gene copies was able to produce, in the best fermentation conditions, 0.43 ​g/L using glycerol as sole carbon source. This work demonstrates that relatively simple pathway engineering in *Y. lipolytica* can lead to significant amount of resveratrol, which most likely can be further improved by manipulating the upstream part of the pathway and the availability of precursors. These results seem to also suggest that *Y. lipolytica* can be a promising host to produce compounds of interest derived from the shikimate pathway.

## CRediT authorship contribution statement

**Qin He:** Investigation, Formal analysis, Validation, Visualization, Writing - original draft. **Patrycja Szczepańska:** Investigation, Formal analysis, Validation, Methodology, Writing - review & editing. **Tigran Yuzbashev:** Investigation, Methodology. **Zbigniew Lazar:** Resources, Supervision, Investigation, Writing - review & editing. **Rodrigo Ledesma-Amaro:** Conceptualization, Resources, Supervision, Investigation, Writing - review & editing.

## Declaration of competing interest

On behalf of the editors I declare that there is no conflict of interest for this article.
