# Supplementary material for: De novo production of resveratrol from glycerol by engineering different metabolic pathways in Yarrowia lipolytica
Source: Metab Eng Commun. 2020 Sep 19;11:e00146. doi: 10.1016/j.mec.2020.e00146 (PMC7522117; doi:10.1016/j.mec.2020.e00146)
Supplement: Multimedia component 1 [file mmc1.docx]

**De novo production of resveratrol from glycerol by engineering different metabolic pathways in *Yarrowia lipolytica***

Qin He^1,2,±^, Patrycja Szczepańska^1,3, ±^, Tigran Yuzbashev^1^, Zbigniew Lazar^3^, Rodrigo Ledesma-Amaro^1, *^

^1^ Department of Bioengineering and Imperial College Centre for Synthetic Biology, Imperial College London, London SW7 2AZ, UK

^2^ Department of Microbiology, Key Lab of Microbiological Engineering of Agricultural Environment, Ministry of Agriculture, College of Life Sciences, Nanjing Agricultural University, Nanjing 210095, PR China

^3^ Department of Biotechnology and Food Microbiology, Wroclaw University of Environmental and Life Sciences, Chelmonskiego 37, 51-630 Wroclaw, Poland

^±^These authors contributed equally to this work

* Correspondence to Rodrigo Ledesma-Amaro. Email: r.ledesma-amaro@imperial.ac.uk.

**Table S1** List of primers used in the study

| **Name** | **Sequence (5’-3’)** |
| --- | --- |
| pZUA2.3/FjTAL/4CL/VvVST-F  pZUA2.3/FjTAL/4CL/VvVST-R | GAATGAACACCATCAACGAGTAC  CACAGACACTAGATTAGTTGGTCACG |
| pBUA2.2/4CL/VvVST-F_1_  pBUA2.2/4CL/VvVST-F_2_  pBUA2.2/4CL/VvVST-R_1_  pBUA2.2/4CL/VvVST-R_2_ | GACGTGATCTTCCGATCTAAG  GTCTATCCCCAAGGCTCCCTC  CGGATAGAGGACAGGTCGTAC  CGATAGACACGTCGGGGTCA |
| pZLA2.2/PAL/C4H-F_1_  pZLA2.2/PAL/C4H-F_2_  pZLA2.2/PAL/C4H-R_1_  pZLA2.2/PAL/C4H-R_2_ | CCTTCTGTGTGTCTGACCCC  AGGACTTCGACAAGGTGTTC  TAGACTTGGTGGAAGCTCGG  TGTCCTCGTTAATCTCTCCC |
| pZLA 2.2/PAL/C4H-F :2 copy  pZLA2.3/FjTAL/4CL/VvVST-F :2 copy  pZLA 2.2/PAL/C4H-R :2 copy  pZLA2.3/FjTAL/4CL/VvVST-R :2 copy | GGCGGGCTTCATGTTAAGAG  GGCGGGCTTCATGTTAAGAG  GAAAGGTAATTCGGGGACGG  GAAAGGTAATTCGGGGACGG |
| pBUA2.2/4CL/VvVST-F :2 copy  pZUA2.3/FjTAL/4CL/VvVST-F :2 copy  pBUA2.2/4CL/VvVST-R :2 copy  pZUA2.3/FjTAL/4CL/VvVST-R :2 copy | GCTCAATGGTCTGCTTGGAG  GCTCAATGGTCTGCTTGGAG  GAAAGGTAATTCGGGGACGG  GAAAGGTAATTCGGGGACGG |

**Table S2** List of plasmids used in the study

| **Plasmids** | **Genotype** |
| --- | --- |
| pYTK001/FjTAL | FjTAL |
| pYTK001/4CL1 | 4CL1 |
| pYTK001/VvVST | VvVST |
| pYTK001/PAL | PAL |
| pYTK001/C4H | C4H |
| pZUS1.1/FjTAL | pTEF-FjTAL, U^+^/L^-^ |
| pZUS1.2/4CL1 | pTEF-4CL1, U^+^/L^-^ |
| pZUS1.3/VvVST | pTEF-VvVST, U^+^/L^-^ |
| pZUS1.1/4CL1 | pTEF-4CL1, U^+^/L^-^ |
| pZUS1.2/VvVST | pTEF-VvVST, U^+^/L^-^ |
| pBUS1.1/PAL | pTEF-PAL, U^+^/L^-^ |
| pBUS1.2/C4H | pTEF-C4H, U^+^/L^-^ |
| pBUA2.2/4CL1/VvVST | pTEF-4CL1, VvVST*,* U^+^/L^-^ |
| pZLA2.2/PAL/C4H | pTEF-PAL, C4H, U^+^/L^-^ |
| pZUA2.3/FjTAL/4CL1/VvVST | pTEF-FjTAL, 4CL1, VvVST, U^+^/L^-^ |

**Table S3** *Y. lipolytica* strains constructed for resveratrol production

|  | **Strain** | **Parent strain** | **Genotype** |
| --- | --- | --- | --- |
| Tyrosine pathway | T | Pold | p*TEF*-*FjTAL*, p*TEF*-*4CL1*, p*TEF*-*VvVST*, Ura^+^, Leu^-^ |
| Phenylalanine pathway | P | Pold | p*TEF*-*PAL*, p*TEF*-*C4H*, p*TEF*-*4CL1*, p*TEF*-*VvVST*, Ura^+^, Leu^+^ |
| Double copy | T2 | Po1d | p*TEF*-*FjTALx2*, p*TEF*-*4CL1x2*, p*TEF*-*VvVSTx2*, Ura^-^, Leu^+^ |
|  | P2 | Po1d | p*TEF-PALx2*, p*TEF*-*C4H x2*, p*TEF*-*4CL1x2*, p*TEF*-*VvVSTx2*, Ura^+^, Leu^+^ |
|  | T2P2 | Po1d | p*TEF*-*FjTALx2*, p*TEF-PALx2*, p*TEF*-*C4H x2*, p*TEF*-*4CL1x2*, p*TEF*-*VvVSTx2*, Ura^+^, Leu^+^ |

Fig. S1 Biomass of strain T and P using glucose media

with and without addition of amino acids

Fig. S2 Biomass of strain T2, P2 and T2P2 using glucose media

with and without addition of amino acids

Fig. S3 Biomass of recombinants using glycerol

with and without addition of amino acids

Fig. S4 Glycerol consumption under different fermentation conditions in bioreactor

Fig. S5 Biomass production under different fermentation conditions in bioreactor

Fig. S6 Biomass, glycerol consumption and resveratrol production by strain T2P2 using glycerol as a substrate in bioreactor under condition 3 (without amino acids added and normal aeration)
